# Unravelling the Therapeutic Potential of Nano-Delivered Functional Foods in Chronic Respiratory Diseases

**DOI:** 10.3390/nu14183828

**Published:** 2022-09-16

**Authors:** Dvya Delilaa Clarence, Keshav Raj Paudel, Bikash Manandhar, Sachin Kumar Singh, Hari Prasad Devkota, Jithendra Panneerselvam, Vivek Gupta, Nitin Chitranshi, Nitin Verma, Sonia Saad, Gaurav Gupta, Philip Michael Hansbro, Brian Gregory Oliver, Thiagarajan Madheswaran, Kamal Dua, Dinesh Kumar Chellappan

**Affiliations:** 1 School of Postgraduate Studies, International Medical University (IMU), Kuala Lumpur 57000, Malaysia; 2 Centre for Inflammation, Centenary Institute and University of Technology Sydney, Faculty of Science, School of Life Sciences, Sydney, NSW 2007, Australia; 3 Discipline of Pharmacy, Graduate School of Health, University of Technology Sydney, Sydney, NSW 2007, Australia; 4 Australian Research Centre in Complementary and Integrative Medicine, Faculty of Health, University of Technology Sydney, Sydney, NSW 2007, Australia; 5 School of Pharmaceutical Sciences, Lovely Professional University, Jalandhar-Delhi G.T. Road, Phagwara 144411, India; 6 Graduate School of Pharmaceutical Sciences, Kumamoto University, Kumamoto 862-0973, Japan; 7 Pharmacy Program, Gandaki University, Pokhara 33700, Nepal; 8 Department of Pharmaceutical Technology, School of Pharmacy, International Medical University, Kuala Lumpur 57000, Malaysia; 9 Macquarie Medical School, Faculty of Medicine, Health and Human Sciences, Macquarie University, North Ryde, Sydney, NSW 2109, Australia; 10 Chitkara School of Pharmacy, Chitkara University, Atal Nagar 174103, India; 11 Faculty of Medicine and Health, The University of Sydney, Camperdown, NSW 2006, Australia; 12 School of Pharmacy, Suresh Gyan Vihar University, Jaipur 302017, India; 13 Department of Pharmacology, Saveetha Dental College, Saveetha Institute of Medical and Technical Sciences, Saveetha University, Chennai 600077, India; 14 Uttaranchal Institute of Pharmaceutical Sciences, Uttaranchal University, Dehradun 248007, India; 15 Woolcock Institute of Medical Research, University of Sydney, Sydney, NSW 2006, Australia; 16 School of Life Sciences, Faculty of Science, University of Technology Sydney, Sydney, NSW 2007, Australia; 17 Department of Life Sciences, School of Pharmacy, International Medical University, Kuala Lumpur 57000, Malaysia

**Keywords:** functional foods, nanoparticles, respiratory diseases, inflammation, pulmonary

## Abstract

Chronic inflammation of the respiratory tract is one of the most concerning public health issues, as it can lead to chronic respiratory diseases (CRDs), some of which are more detrimental than others. Chronic respiratory diseases include chronic obstructive pulmonary disease (COPD), asthma, lung cancer, and pulmonary fibrosis. The conventional drug therapies for the management and treatment of CRDs only address the symptoms and fail to reverse or recover the chronic-inflammation-mediated structural and functional damage of the respiratory tract. In addition, the low efficacy and adverse effects of these drugs have directed the attention of researchers towards nutraceuticals in search of potential treatment strategies that can not only ameliorate CRD symptoms but also can repair and reverse inflammatory damage. Hence, there is a growing interest toward investigating the medicinal benefits of nutraceuticals, such as rutin, curcumin, zerumbone, and others. Nutraceuticals carry many nutritional and therapeutic properties, including anti-inflammatory, antioxidant, anticancer, antidiabetic, and anti-obesity properties, and usually do not have as many adverse effects, as they are naturally sourced. Recently, the use of nanoparticles has also been increasingly studied for the nano drug delivery of these nutraceuticals. The discrete size of nanoparticles holds great potential for the level of permeability that can be achieved when transporting these nutraceutical compounds. This review is aimed to provide an understanding of the use of nutraceuticals in combination with nanoparticles against CRDs and their mechanisms involved in slowing down or reversing the progression of CRDs by inhibiting pro-inflammatory signaling pathways.

## 1. Introduction

For over hundreds of years before the existence of modern medicine, natural products were used to maintain the health and wellbeing of humans. Back in the day in regions of China and India, the knowledge of herbs and herb-related remedies were passed down through generations to help overcome the symptoms and pathologies of diseases and health issues, including respiratory diseases. In general, edible and medicinal herbs are used in local cuisines to provide added health benefits. With the progression of science and technology over the past several years, there has been an increase in the scientific understanding of the diet and benefits to the general health and wellbeing of humans, and this has progressed to the development of a category of food called functional foods. The term ‘functional food’ means foods that have a variety of components with nutritional and medicinal value and can reduce the risk of diseases and maintain wellbeing. They can be divided into two categories: conventional and modified functional foods. Conventional functional foods include natural or whole-food components with beneficial properties. On the other hand, modified functional foods are food products with added ingredients for targeted health benefits. Functional foods can range from fruits, vegetables, and grains to several other types of food sources with sufficient quantities of beneficial properties. They can also consist of foods that have dietary fiber, polyunsaturated fatty acids, probiotics, and so on that can be used for various types of health issues and diseases [1]. Over the recent years, the concept and study of functional foods have grown exponentially, and this has resulted in an abundance of available scientific information regarding specific foods and their health benefits.

It is estimated that 20–30% of modern drugs that are prescribed worldwide today are sourced from plants. These plant-derived active biomolecules are mostly obtained in the form of polyphenols, flavonoids, terpenes, and several other classes of plant compounds. They are proved to be beneficial in protecting against diseases and preventing nutrient deficiencies, as well as promoting proper growth and development. Additionally, studies have shown that many natural products are used to treat various types of health issues. This is mainly due to the fact that many functional foods have properties such as anti-inflammatory, anticancer, antioxidant, and protective gastrointestinal properties, amongst others. There is evidence that suggests that the dietary consumption of functional foods can be linked to a reduced risk of chronic diseases. For example, certain plant-based foods, including fruits, vegetables, herbs, cereals, nuts, and beans, have high contents of vitamins, minerals, antioxidants, fiber, omega-3 fatty acids, and phenolic compounds, which play a protective role against chronic diseases, such as cancer, cardiovascular and gastrointestinal-related disease. Additionally, other foods, including dairy products, eggs and seafood, have beneficial properties; for example, fish contains omega-3 fatty acids (eicosapentaenoic acid and docosahexaenoic acid). These important compounds play a key role in the development of the heart and brain health.

The lungs are a very important organ in the human body. The inhalation of tobacco smoke, toxic chemicals, or environmental pollutants and occupational irritants can cause airway inflammation, a persistent manifestation of which may result in several chronic inflammatory lung diseases, such as chronic obstructive pulmonary disease (COPD), asthma, cystic fibrosis, and lung cancer. A high level of reactive oxygen species in the lungs may cause damage to the respiratory tract, which can result in hypoxia, inflammation, tissue injury, airflow obstruction, and a decline in the forced expiratory volume [2]. The progression of such inflammatory conditions can worsen and become irreversible, as well as become detrimental to a patient’s wellbeing. In most respiratory diseases, generally, there is a low level of antioxidant defense; therefore most patients are usually advised to increase the intake of foods with antioxidant and anti-inflammatory properties in order to increase the contractile ability of the lungs, as well as to soothe inflammatory responses.

Although numerous studies and research works have suggested the promising effects of functional foods, most functional foods have been unsuccessful in the clinical drug development stages, mainly because the majority of these foods have low oral bioavailability, which is rooted in poor aqueous solubility and absorption [3]. Many functional food components are highly water-soluble, and as a result, they demonstrate poor absorption, as they are unable to cross lipid membranes. They also have large molecular sizes, which results in low bioavailability and efficacy. Drug solubility and permeability characteristics during drug development are two key points that allow control and a better rate of drug absorption. Therefore, to address this gap, in-depth studies need to be conducted using multiple approaches to enhance these characteristics, such as chemical modification, salt formation, amorphization, particle size reduction, or different formulation development strategies, including solid dispersions, molecular complexation [4], lipid-based formulations, micelles [5], and nanoparticles [6]. These can, in turn, produce a more effective and safer drug that can potentially address various health conditions and diseases.

Over the recent decades, nanotechnology has been used in a vast majority of industries for a variety of research purposes. This is mainly due to its unique and extraordinary properties, which include its minute nanoscale that has truly revolutionized the usage of it. Additionally, nanoparticles (NPs) are favorable for various types of studies due to their tunable physicochemical characteristics, including their melting point, electrical and thermal conductivity, catalytic activity, wettability, light absorption, and scattering results [7]. Specifically looking at the potential in medicine and pharmaceuticals, the use of nanotechnology has been proved to provide a vast number of benefits to treat and overcome many diseases. This is mainly because of the increased ability to provide an alternative drug and delivery system, biomedical imaging, and therapeutics for various issues. The common forms of nanotechnology-based formulations currently being used include nanoemulsions, nanoliposomes, carbon nanotubes, nanoencapsulations, and nanomicelles, amongst others. There are NPs that tend to show little or no side effects when delivering drugs to the target site.

Generally, NPs ranges from 1–100 nm in diameter. Their definition varies depending on their use for specific references by different organizations. However, recently the British Standards Institution provided a guideline to help identify different concepts in nanotechnology, including terms such as nanoscale, nanoscience, nanotechnology, nanomaterial, nano-object, nanoparticle, nanofiber, nanocomposite, nanostructure, and nanostructured materials, amongst others [8]. Nanomaterials can be classified into four material-based categories, being carbon-based nanomaterials and inorganic-based nanomaterials, as well as composite-based nanomaterials.

NPs are currently being studied as a delivery system to encapsulate and deliver functional foods to target disease sites. The use of nanotechnology in science and medicine has been an active area of research over the past several years. Additionally, they have been used therapeutically to deliver small molecules of drugs, peptides, proteins, and nucleic acids. The use of NPs, in general, has proved much more beneficial compared to other delivery systems, as they have advanced pharmacological and physiochemical characteristics, such as melting point, electrical conductivity, light absorption, and scattering, which in turn have resulted in much better performances in comparison to their bulk counterparts [7]. However, the optimum use of NPs in drug delivery can be altered using multiple factors, including size and surface properties, which influence the pharmacokinetics and pharmacodynamics of a system.

The concept of combining nanotechnology and functional food is something that has been part of food processing for many years, as most foods present themselves naturally in a nanoform. The understanding of the term nanofood means foods or commonly edible substances that are cultivated, manufactured, and packaged through systems that use nanotechnology or are produced with added nanomaterials [9]. The concept of nanofood has proved to be purposeful by allowing companies to cut cost, improve the safety of food substances, and increase the taste and beneficial nutrition of foods that are perishable or semiperishable in nature. As we know, many food substances that contain potent bioactive substances tend to have low potency, mainly because they have low stability in the gut and reduced retention time in the intestine, as well as low permeability to body cells. With the help of NPs and their encapsulating nature, as well as their large surface area and decreased particle dimension, bioactive compounds are able to overcome their limitations [10].

Various types of NPs have been studied for their potential use as functional drug delivery systems for medication. Currently, research is continuously being conducted to identify the potential uses of NPs for the delivery of functional foods. This is mainly because NPs have many beneficial properties that could be useful for optimizing the delivery of functional foods by encapsulation, protection, and controlled release. Functional foods are preferred when using nanoparticles, as they have more advantageous properties, such as the ability to deliver more than one active constituent using the same carrier, increased bioavailability, better targeting, increased residence time in the body, sustained release systems, and reduced side effects.

## 2. Inflammation in the Lungs

The lungs are specialized organs for respiration and, thus, constantly interact with the external environment through inhaled air. Often, they come into contact with various types of stimulants, including chemicals, allergens, toxins, pollen, and dust, as well as foreign antigens, that can cause inflammation and infection of the respiratory tract [11]. The lungs carry out their function of pulmonary respiration by having a gentle diffusion capacity. There are several methods and indicators of how healthy the lung and respiratory demands of everyone should be from person to person. Of these indicators, forced expiratory volume (FEV) and forced vital capacity (FVC) are the main methods to identify the function of the lungs amongst individuals [12]. Another method of measurement that is usually only studied in animal models is the determination of levels of crucial physiological indicators, such as glutathione, glutathione peroxidase, and catalase, as well as the contents of malondialdehyde and hydroxyproline found in the lungs.

Asthma and chronic obstructive pulmonary disease (COPD) are common forms of chronic respiratory diseases characterized by chronic inflammation. COPD is the third-highest leading cause of deaths worldwide [13,14]. These conditions affect the livelihoods of individuals suffering worldwide [15,16], with an approximate of 235 million suffering from asthma and 3 million lives lost to COPD each year [17]. COPD is mostly prevalent in smokers and individuals aged 40 and above, as cigarette smoke on average has around 4500 types of toxins, carcinogens, mutagens, and heavy metals that usually are deposited throughout different areas of the lungs and eventually cause the destruction of respiratory tissue [18,19]. With the alarming increase in the rate of inflammatory disease incidences due to issues such as worsening air pollution, increased occupational allergens, and the increase in smoking population [20,21], there is an urgent and crucial need to discover new and innovative remedies to promptly manage these issues.

Chronic inflammatory respiratory diseases often present themselves with symptoms such as wheezing, cough, dyspnea, and increased chest wall diameter and sputum production, amongst others [22]. Individuals with COPD usually develop clinical symptoms after many years after smoking or being in a constant environment exposed to similar irritants. COPD is characterized by chronic lung inflammation that can result in progressive and irreversible airway hyperresponsiveness, mucus hypersecretion, and airflow obstruction, as well as airway inflammation and airway remodeling [23], with the potential for worsening and exacerbations. The key feature of this form of inflammation that causes obstructions in the airflow is mainly the combination of emphysema with chronic bronchitis [24]. Chronic bronchitis, which is another serious inflammatory condition of the lungs, is caused by the narrowing of the bronchial tubes, as well as inflammation due to a lack of cilia, which causes excessive mucus secretion. The inflammation in the airway is also commonly accompanied by other cardiovascular diseases (CVDs) and lung cancer.

Emphysema is one of the most common physiological changes observed in COPD patients. This can be categorized by the destruction of the alveolar air sacs by the neutrophils and macrophages in the body when irritants disrupt and cause an inflammatory response. The small-sized alveoli located in the lungs are found to swell and then reduce the absorption capacity and, in turn, result in tissue destruction and impaired gas exchange [25]. Concurrently, there is also protease-mediated destruction, which causes the loss of elastic recoil and, over a long period of time, results in the collapse of the airway when an individual exhales [26]. Additionally, the obstruction to the airways, as well as the inflammatory response, result in a decrease in the FEV. Patients with COPD also tend to show hyperinflation of the lungs, mainly caused by trapped air from collapsed airways during the process of exhalation. The collapsed airways also disrupt the ability of CO_2_ to be fully expelled from the body, leading to CO_2_ retention. In addition, acute exacerbation is also common in COPD patients, which is associated with increased inflammation [24].

**Figure 1:**
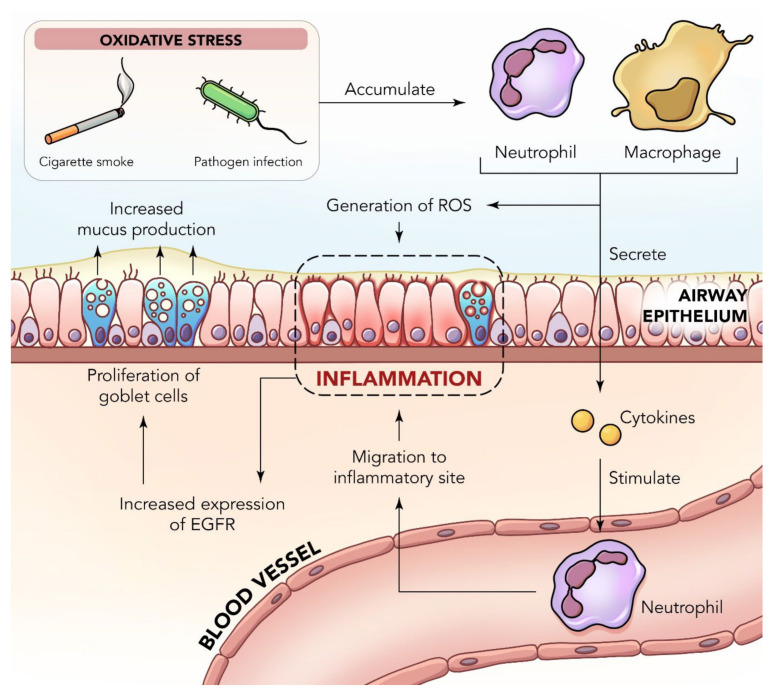
The mechanistic pathways leading to the pathogenesis of inflammatory lung diseases. ROS—Reactive Oxygen Species; EGFR—Epidermal Growth Factor Receptor.

Inflammation is an extremely important homeostatic cellular response, as well as a host defense mechanism, that helps protect the lungs against harmful foreign substances. Inflammation is often classified as acute or chronic. Acute inflammation is an initial response, which can be identified by resident cell activation and the release of proinflammatory cytokines, as well as chemokines, culminating in the recruitment of polymorphonuclear particles, mainly neutrophils, which are sourced from the innate immune system to the injury site. This response is usually complex and promotes cardinal signs of inflammation, including pain, heat, and edema [27]. Chronic and persistent inflammation is a prolonged response that is characterized by a gradual change in the cell types found at the inflammatory site, and over a long period of time, it can cause damage that is permanent [28]. However, in both instances of inflammation, there is a prevalence of vasodilation, increased local blood flow, liberation of pro-inflammatory mediators, and fluid extravasation [29,30]. Chronic inflammation causes airway narrowing and decreased lung recoil, as well as other serious diseases, and in severe respiratory failure leads to death (Figure 1). The mechanisms underlying the incidence and progression of COPD are complicated and include several pathogenic factors, such as the imbalance of oxidation–antioxidation, inflammatory reaction, cell apoptosis, and glucocorticoid resistance [31]. In fact, oxidative stress resulting from cigarette smoke was found to trigger autophagy impairment in COPD emphysema [32].

The progression of inflammatory lung diseases begins with the body’s innate immunity [33], whereby the toxic gases from cigarette smoke and the external environment activate the pattern recognition receptors, as well as the purinergic receptors. Together with these receptors, there are additional necrotic apoptotic cells that contribute to disease progression by releasing damage-associated molecular patterns and commencing the inflammatory response [34,35]. In addition, the gases produced by cigarette smoking cause the inhibition of vascular endothelial growth factor (VEGF), as well as hepatocyte growth factor, which damage the epithelial cells and cause the apoptosis of alveolar cells, resulting in emphysema. With the alveolar epithelial cells in a damaged state, the body then releases tissue growth factor (TGF)-β via the connective-TGF, which causes local fibrosis, collagen deposition, and airway remodeling [18,35].

Environmental irritants can activate the alveolar surface macrophages [36] and airway epithelial cells to release chemotactic factors, including chemokine ligand 2 (CCL2). These factors then attract other components, including the monocytes, C-X-C motif ligand (CXCL)-1, and CXCL-8. In turn, these compounds then attract neutrophils, CXCL-9, CXCL-10, and CXCL-11. Lastly, these compounds then attract Th1 and cytotoxic T cells [34]. The alveolar macrophages have an additional function whereby they are recruited as feedback to the chemokines CCL2 and CXCL-1. This contributes to the damage of the alveolar tissue by the production of reactive oxygen species (ROS), matrix metalloproteinase (MMP)-9, and the cathepsins K, L, and S. Additionally, there is the production of granulocyte, which is secreted by the lung macrophages and assisted by the granulocyte-macrophage colony stimulating factor, which then results in increased granulocyte and neutrophil circulation [35] Other components, such as leukotriene B4, CXCL-1, CXCL-5, and CXCL-8, also have the ability to cause the migration of neutrophils towards the affected area, which soon after causes the destruction of alveoli and increases the production and secretion of mucus through the airway of goblet cells [18,35].

**Figure 2:**
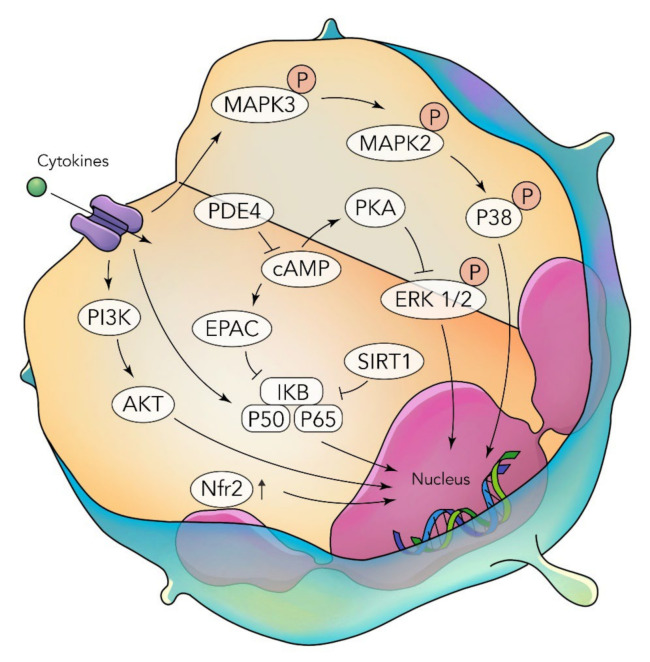
Cytokine-mediated mechanistic pro-inflammatory pathways in a neutrophil [51].

When inflammation is triggered in the lungs in COPD, it prompts the activation of inflammatory cells, including CD8+ lymphocytes, macrophages, eosinophils, and neutrophils in the peripheral spaces (Figure 2). The activation of these cells then results in the production of cytokines and various inflammatory mediators that advance the inflammatory response and tissue remodeling, such as interleukin (IL)-6 and tumor necrosis factor (TNF)-β [37]. Some of the other cytokines and mediators that promote inflammation are IL-5, IL-1β, IL-13, leukotrienes, prostaglandins, histamines, and TNF-α [28]. Additionally, the nuclear factor (NF)-κB, cyclooxygenase-2 (COX-2), and inducible nitric oxide synthase (iNOS) pathways are also crucial in the pathogenesis of inflammation [38]. The free radicals, superoxide radicals, and lipopolysaccharide (LPS) found in cigarette smoke can begin the onset of transcription factor NF-κB. The NF-κB signaling pathway is a key part of the immune response, as it is essential to inflammatory processes due to its importance in the transcription of cytokines, such as TNF-α, IL-1β, IL-6, and nitric oxide (NO) [39], as well as its important role in the airway remodeling action of COPD. COX-2 is an inducible enzyme that is naturally regulated by the NF-κB pathway and induced transcriptionally by many other stimuli with a crucial function in the pathogenesis of airway inflammation [40]. COPD causes an increase in inflammatory markers and mediators, including TNF-α, IL-6, and C-reactive protein (CRP), to name a few [41], and can cause other forms of complications, including lung cancer, stroke, cardiovascular diseases, atherosclerosis, and myocardial ischemia [42]. With that in mind, many studies have targeted research on cytokines and inflammatory markers in order to prevent the worsening of these diseases [43].

Although there has been a lot of advancement in science and medicine, there is still a large gap in the efforts to successfully manage and cure inflammatory lung diseases [28,44,45]. Current methods of therapy include using high doses of more than one type of therapeutic drug, which can cause adverse side effects to patients. Previously, potent steroidal hormones or nonsteroidal anti-inflammatory drugs (NSAIDs) have been used as anti-inflammatory drugs. However, they have limitations, such as the body being able to easily develop tolerance toward them, as well as damaging effects to the gastrointestinal tract [46]. The use of corticosteroids, phosphodiesterase (PDE) inhibitors, long-acting β_2_ agonist, and anticholinergics also produces additional adverse effects, such as the suppression of the hypothalamic–pituitary adrenal axis, slowed growth and collagen production in children, supraventricular tachycardias, acute glaucoma, myocardial ischemia, electrolyte disturbance, arrhythmias, myocardial infarction, and osteoporosis [47,48,49], which in turn increase susceptibility to other types of diseases [28,50]. Therefore, it is abundantly clear that there is still an unmet need for developing a remedy that allows patients to have an improved quality of life despite their circumstances. Thus, it is extremely crucial that further studies and research go into the development and innovation of a new, effective, and safe alternative that can address the issue of increasing morbidity and death related to COPD.

## 3. Functional Foods in the Management of Inflammatory Lung Disease

Functional foods, better known as nutraceuticals, are a combination of nutrients and pharmaceuticals. Various types of diets and nutrition have been studied, and it has been found that different foods play important roles in the prevention of diseases in general. Apart from the medical benefits, these functional foods also have nutritional value that aids in the maintenance of a healthy body while also regulating body functions [52]. These functional foods are also vigorously being investigated, as they have many beneficial properties, including the potential to reduce the overall cost of pharmaceutical production since they can be found naturally. In general, this can allow pharmaceutical companies to mass-produce treatments at low costs, making them affordable to patients in low-income countries [53]. In addition to this, since these products are sourced naturally, they tend to show a reduced amount of side effects and adverse effects in comparison to chemically synthesized medications [54,55].

Studies over the past several years have indicated the importance of proper dietary intake in obstructive lung diseases, such as asthma and COPD. These studies have taken into consideration both early life and disease development, as well as the management of disease progression [56,57]. As highlighted earlier in this review, respiratory diseases can be identified by having airway and systemic inflammation, reduced lung function, ROS overproduction, airflow obstruction, and significant higher chances of morbidity and mortality, as well as indirectly causing severe economic burdens. Additionally, issues such as fibrosis, inflammatory responses, and mucus deposition can cause irreversible damage to a patient, mainly because when an individual has a respiratory disease, they usually have extremely low antioxidant ability [58].

**Figure 3:**
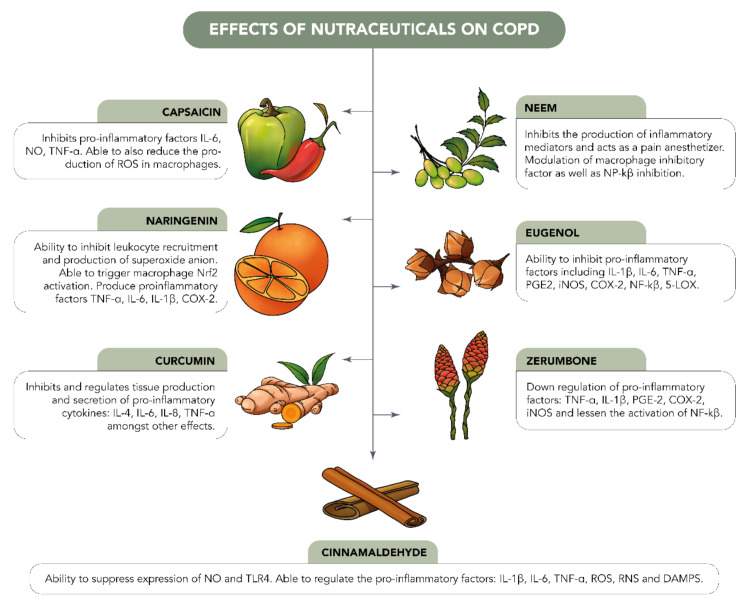
The effects of various nutraceuticals on lung inflammation and COPD.

Functional foods can contribute to the prevention and management of inflammatory lung diseases (Figure 3). Many of them have beneficial properties, including antioxidant, anticancer, anti-obesity, and (most importantly for lung diseases) anti-inflammatory properties. Some examples of common nutraceuticals include curcumin, green tea, caffeine, eugenol, and so on. The antioxidant activity of these components can be utilized to counteract against the overproduction of ROS. There are also other functional foods that have strong anti-inflammatory activities and can be further studied to target inflammation in lung diseases. Additionally, epigallocatechin gallate (EGCG), considered one of the most important nutritional elements found in green tea, plays a protective role in pulmonary fibrosis by inhibiting chemoattractant, as well as managing inflammatory responses in several fibrotic diseases.

Several studies have in the recent past reported on the therapeutic effects of functional foods for chronic respiratory diseases. In one study, the relaxant effect of the functional food berberine was investigated on the tracheal smooth muscle of a guinea pig. The results showed that berberine was able to relax the carbachol-induced precontraction of the tracheal muscle in a concentration-dependent manner due to its effect on the muscarinic acetylcholine receptors. These positive results prove that this functional food could be beneficial in the treatment of chronic respiratory diseases, as many such patients commonly face symptoms that strain the tracheal smooth muscles [59]. In another study, the effect of *Alstonia scholaris* was tested against rat lipopolysaccharide (LPS)-induced airway inflammation. The results obtained show that there was a decrease in the white blood cells, as well as in the inhibition of IL-8 and TNF-α. These collaborative effects also proved to reduce the amount of tissue damage in the lungs [60].

Significant contributions have been reported in the literature with much emphasis on functional foods that are therapeutically active on the lung epithelium. Such foods that are found in nature have been demonstrated to exert protection and healing, largely through the potent nutrients present within them. Molecular-level mechanisms have been demonstrated for several of these natural biomolecules. Apart from the bioactive functional foods described above, there are also several other beneficial compounds, such as curcumin, celastrol, rutin, quercetin, naringenin, and theaflavins, that have also been found to have anti-inflammatory properties having beneficial effects on airway mechanisms. These compounds show anti-inflammatory activity by interfering with the inflammatory pathway, activator protein 1, and the NF-kB pathway. There have been reports on the molecular mechanisms of such nutrients where they interfere with cell communication networks and interact with various receptors found along the respiratory tract, eventually bringing out their biological actions. These compounds are also known to neutralize harmful toxins and free radicals that are generated in the body, thereby providing a synergistic action. Many functional foods also consist of flavonoid compounds, which are also known for their anti-inflammatory properties. One of the beneficial properties of flavonoids is their ability to reduce the production and expression of extracellular matrix (ECM) genes. Overall, flavonoids have the crucial role of overseeing several pathways in the human body [61]. However, there is another compound that plays the role of modulating flavonoids, and that is quercetin [62]. Quercetin carries out its function by interrupting the redox balance found in pulmonary fibrosis, and this is conducted via the modulation of the nuclear factor-erythroid factor 2-related factor 2 (Nrf2) pathway. Quercetin carries out its function by increasing the activity of the Nrf2 action, resulting in an increase in antioxidant response [63]. In a nutshell, the increased uptake of fruits and vegetables in the human diet is able to intensify the amount flavonoids, as well as to reduce the expression of pro-inflammatory cytokines, including IL-8 and TNF-α. Functional foods have promising effects on COPD, as most of them target mediators including IL-6, IL-8, IL-1β, TNF-α, COX-2, and PGE-2, amongst others.

### 3.1. Zerumbone

Zerumbone, (2E, 6E, 10E)-2,6,9,9-tetramethylcycloundeca-2,6,10-trien-1-one) is a natural, cyclic, eleven-membered, crystalline sesquiterpene present in rhizomes of Zingiber zerumbet, *Zingiber montanum*, and many other species of the Zingiberaceae family. Zingiber zerumbet ginger is usually distributed around locations where the climate is categorized to be hot and humid throughout the year in countries such as India, Malaysia, Indonesia, Bangladesh, Hawaii, China, and Thailand. Throughout the many years of its application, it has been commonly and widely used as spices, flavoring agents, and medicines. This plant is widely found throughout southeast Asia due to its preferential growth in tropical climates.

Zerumbone is highly lipophilic, and the poor water solubility contributes to poor absorption, low oral bioavailability, and limited targeting to tissues and organs of interest [64,65]. Zerumbone has been found to possess many medicinal properties and has been used extensively in traditional medicine to treat fever, inflammation, toothache, indigestion, constipation, diarrhea, ulcers, diabetes, asthma, rheumatism, stomach pain, skin diseases, and several other conditions. Multiple reviews have demonstrated the beneficial pharmacological effects of zerumbone for different diseases, such as various types of cancer, wound healing, obesity, and inflammatory bowel diseases, amongst others. This has been confirmed by several studies on zerumbone extracts, which have found properties such as anti-inflammatory, antibacterial, antiplatelet, antifungal, cytotoxic, and chemopreventive properties, as well as several other beneficial properties [66].

**Figure 4:**
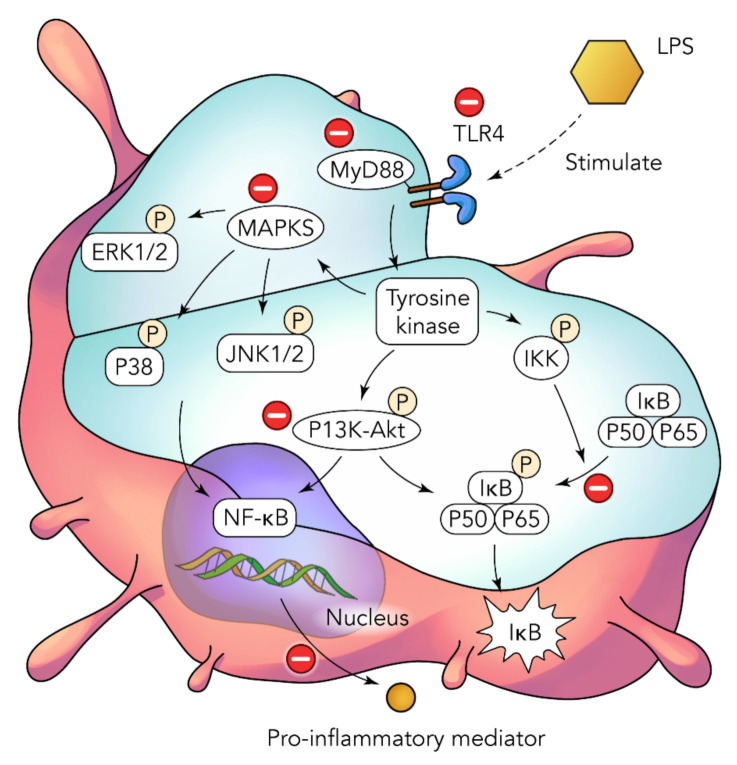
The schematic illustration of the potential mechanism of zerumbone in the suppression of LPS-stimulated inflammatory response in a macrophage [66].

One of the key properties of zerumbone that has an amplitude of potential is its anti-inflammatory activity. In human lymphocytes, the cytotoxic effects of the major pro-inflammatory cytokines TNF-α and IL-1β are crucial mediators that carry out important function during the propagation of various inflammatory conditions, such as lung injuries. Zerumbone has been found to have effectiveness against various inflammatory and immune conditions by molecularly targeting and downregulating various pro-inflammatory factors found in LPS-induced human macrophages (Figure 4), such as TNF-α, IL-1β, PGE-2, and COX-2. Based on Figure 4, it can be understood that zerumbone has the potential and ability to inhibit the activation of NF-κB by the phosphorylation of Akt, as well as the ability to suppress I3K-Akt and MAPK signaling. The diagram also elucidates the various MAPKs, which include ERK1/2, p38 and JNK1/2, which are all from the family of serin/threonine protein kinases. This group of protein kinases plays a crucial role in regulating a vast range of cellular activities, and any form of upregulation can result in many types of health issues, including inflammation and cancer, amongst others. With that in mind, the downregulation of these kinases is beneficial in preventing the release of inflammatory factors. Additionally, when inflammation is induced through LPS, the TLR4 protein receptor is triggered, and it releases signals through the MyD88 adaptor protein to activate the PI3K-Akt, NF-κB, and MAPK pathways. The understanding of this pathway and mechanism helps us predict the molecular target for further studies.

In a previous study, zerumbone downregulated the mRNA expression of TNF-α, and IL-1β was suppressed in a concentration-dependent manner [67]. Additionally, other mediators such as COX-2, also play a crucial role in inflammation, as it allows the conversion of arachidonic acid to PGE2. PGE2 plays a substantial function in the inflammation of the pulmonary system, as well as macrophage polarization in COPD [68]. Zerumbone was found to diminish the expressions of COX-2 and PGE2 in a dose-dependent manner [67].

In one study focusing on acute lung injury (ALI), it was found that the pretreatment of zerumbone reduced LPS-induced pulmonary edema and leukocyte infiltration. In this study, zerumbone was found to inhibit the secretion of TNF-α and IL-6 into the alveolar space of LPS-induced ALI murine models. Additionally, there was also evidence that zerumbone was able to inhibit the expression of pro-inflammatory mediators, iNOS, and COX-2 in LPS-induced ALI. It was also found that zerumbone was able to lessen the activation of NF-κB in a concentration-dependent manner [69].

#### Zerumbone Delivery Using Nanoparticles

Similar to most functional foods, zerumbone has shown promising beneficial activities in vivo and in vitro. However, it has limitations to its pharmaceutical usage due to its poor water solubility and low bioavailability, leading to poor absorption in the body. Such limitations also correlate to having a limited target ability to successfully reach specified tissues and organs [70]. Therefore, factors that may enhance the delivery and absorption of zerumbone have been studied by several researchers.

In a previous study, the in vitro activity of a zerumbone-loaded nanosuspension drug delivery system was evaluated [61]. The nanosuspensions were formed using a high-pressure homogenization process involving sodium dodecyl sulphate (SDS) and hydroxypropylmethylcellulose (HPMC), which acted as stabilizers [64]. This was intended to overcome the poor solubility and dissolution characteristics of zerumbone on its own. The study was conducted for 3 days at various temperatures of 4 °C, 25 °C, and 37 °C [61]. On its own, unprocessed zerumbone in a solution form had a solubility of only 17.85 ± 1.52 µg/mL in comparison to zerumbone in the nanosuspension using SDS or HPMC stabilizers, whereby it was found that the solubility was significantly increased to the values of 26.8 ± 3.88 and 32.11 ± 1.17 µg/mL, respectively [64]. Additionally, the drug release ability of crude zerumbone was found to be 31.92% ± 1.20, whereas the drug release values in nanosuspension form using SDS and HPMC stabilizers were 54.59% ± 7.34 and 75.04% ± 2.17, respectively [61]. This provided a statistically significant result that showed the improved dissolution of zerumbone in a nanosuspension form [64]. Overall, a significantly improved saturation solubility and dissolution profile was observed for the zerumbone nanosuspension. As a result, the increased saturation solubility and dissolution rate could, in turn, provide better drug bioavailability in the body. This indicated that there is an extremely optimistic future for the use of zerumbone as a successful therapeutic agent in the near future.

In another study, zerumbone-loaded nanostructured lipid carriers (ZER-NLCs) were tested in vitro in canine mammary adenocarcinoma cells to evaluate the effects and ability of this functional food to induce apoptosis [67]. As stated previously, zerumbone is a functional food with many promising characteristics, including its anticancer properties. In fact, the findings of this research support the theory that canine mammary gland tumor (CMT) cell apoptosis was induced by zerumbone as a result of the downregulation of the Bcl-2 gene followed by upregulation of Bax gene expression [70]. However, due to its poor water solubility, absorption, and bioavailability, as well as its inefficiency to be delivered to target tissues, it has not been used to its full potential in pharmaceutical drug development [70].

In another study ZER-NLCs with a size averaging at 54.04 ± 0.19 nm were developed, as it is known that NPs of approximately 50–100 nm in size are able to exceed the glomerular capillary threshold of 10 nm [71] while still being considered small enough to avoid being eliminated from the body by immune cells, liver uptake, or clearance from body circulation [72]. It was found that both the ZER-NLCs and zerumbone decreased the proliferation of CMT cells significantly in a time-and-concentration-dependent manner, which indicated that the presence of NLCs did not cause a compromise in the cytotoxic effect of zerumbone on its own. However, it was also observed that the ZER-NLCs were more beneficial in the sense that they were more toxic toward the CMT cells than the zerumbone on its own, and this may have been contributed by the properties of NLCs on their own by their adherence to cell membranes, internalization, and degradation of cellular components [73].

Additionally, the results indicated that the ZER-NLCs induced apoptosis in a slower manner compared to zerumbone on its own. This advocated the idea that zerumbone is released in a sustained manner from ZER-NLCs, which is pharmaceutically beneficial since it allows for a longer therapeutic effect without frequent dosing. Sustained release also aids a prolonged circulation time of zerumbone in the body, which in turn results in improved uptake and accumulation in tumor tissues. Moreover, sustained release using ZER-NLCs would ensure there is no sudden spike of the loaded drug in the blood circulation, thus avoiding the risk of causing acute toxicity in the body [74].

### 3.2. Curcumin

Curcumin, chemically (1E,6E)-1,7- bis(4-hydroxy-3-methoxyphenyl)-1,6-heptadiene-3,5-dione, is a polyphenol compound with two aromatic O-methoxy phenolic groups, a β-dicarbonyl moiety, and a seven-carbon linker containing two enone moieties. It is a dietary, fat-soluble aromatic phyto-extract that is usually characterized by its orange-yellow pigment. It has been extensively explored for its therapeutic potential and is the main compound present in the rhizomes of Curcuma longa from the Zingiberaceae family [75].

Over many years, it has been commonly used as a food additive, a natural coloring agent, and medicine across parts of Asia. Its wide range of usage as a natural drug is enforced due to its ability to treat many pro-inflammatory health issues [76], such as cancer, depression, COPD, obesity, atherosclerotic vascular disease, dementia, diabetes, and others. Additionally, it shows effectiveness against many other disorders, such as lupus nephritis, irritable bowel syndrome, fibrosis, and so on [77]. The many medical properties of curcumin can be summarized by its capabilities and properties of immune modulation, anti-inflammatory, antioxidant, anticancer [78,79] neuroprotective, antidiabetic, cardiovascular protective, and hepatoprotective effects, amongst others [80]. Additionally, certain structural modifications of curcumin using various functional groups have been proved to increase the bioactive properties, as well as the physiochemical availability, of this extract.

Curcumin plays a protective role in COPD by modulating the balance of Th17 and Tregs with pro-inflammatory and anti-inflammatory factors [81]. It has the ability to inhibit and regulate tissue production, as well as the secretion of pro-inflammatory cytokines, including IL-4, IL-6, IL-8, and TNF-α [82,83]. Curcumin has the ability to increase the production of anti-inflammatory cytokine secretion, including IL-10 [Design, synthesis and evaluation of novel diaryl-1,5-diazoles derivatives], as well as soluble intercellular adhesion molecule 1 (sCAM-1) [82,84]. Curcumin ameliorated alveolar epithelial injury in COPD rats by decreasing the levels of interleukin (IL)-6, IL-8, tumor necrosis factor-a, and p66Shc [85,86].

A number of studies have provided evidence of the benefits of curcumin in the treatment of COPD. In one study, the effect of curcumin on airway inflammation in a cigarette-smoking-induced COPD murine model was investigated. Curcumin was found to effectively attenuate airway inflammation and airway remodeling. It significantly reduced the production of neutrophils and lymphocytes, as well as the levels of cytokines. Curcumin was able to suppress the degradation of IκBα and the expression of COX-2 simultaneously, suggesting potential therapeutic benefits of curcumin against COPD.

In another study, curcumin was able to inhibit pro-inflammatory signaling pathways [42]. This included the NF-κB and myeloid differentiation protein 2-TLR 4 co-receptor pathways. Additionally, it inhibited the production of pro-inflammatory mediators, including TNF-α and IL-1β [42]. Curcumin has beneficial effects on the status of various diseases involving chronic inflammation. However, the absorption of curcumin is poor, and even if absorbed into the body, it is rapidly metabolized and excreted in feces. To overcome its low bioavailability, poor absorption, rapid metabolism, and rapid elimination, various drug delivery systems are currently being studied [42]. Interestingly, there is growing evidence that approaches using nanoparticles can resolve the issues of the low bioavailability and biocompatibility of curcumin due to its insolubility in water.

Another study by Tang and Ling investigated the ability of curcumin to ameliorate COPD by modulating autophagy and endoplasmic reticulum stress through the regulation of SIRT1 in rat models [87]. NAD-dependent protein deacetylase sirtuin-1 (SIRT1) is found mainly in the nuclei of cells and plays a key role as a mediator in regulating various processes, including oxidative stress resistance, cellular proliferation, apoptosis, tumorigenesis, endothelial functions, and inflammatory reactions [87]. Additionally, SIRT1 has the ability to act as a positive regulator in autophagy modulation through the process of combining autophagy proteins Atg5, Atg7, and Atg8 to form a molecular complex [88]. In COPD patients, SIRT1 serum levels were found to be much lower [88]. The resulting effect of the decreased SIRT1 was that there was also a suppression and downregulation of FOXO3, which in turn increased NFkB, resulting in pro-inflammatory responses in human bronchial epithelial cells in COPD patients [89,90]. SIRT1 also inhibited the endoplasmic reticulum (ER)-stress-induced apoptosis in cardiomyocytes [91]. Curcumin was found to increase the expressions of SIRT1, LC3-I, LC3-II, and Beclin1, and it decreased the expressions of CHOP and GRP78 in COPD rats [92]. This study suggested that curcumin has high potential to reduce the prevalence of COPD, and further studies can confirm the exact mechanism of this function.

#### Curcumin Delivery Using Nanoparticles

In a study investigating the anti-inflammatory activity of curcumin, silica-containing redox nanoparticles (siRNPs) were used. Curcumin was chosen as a useful extract, as it has anti-inflammatory and antioxidant characteristics that could potentially be used to overcome chronic inflammation diseases caused by the overproduction of ROS. However, similar to most functional foods, curcumin has limitations, which include poor solubility and bioavailability, as well as oxidation caused by ROS. To overcome these limitations, siRNPs were prepared using the self-assembly of an amphiphilic block copolymer consisting of a drug-absorptive silica moiety and a ROS-scavenging nitroxide radical moiety in the hydrophobic segment. This was followed by the encapsulation of curcumin through the dialysis method to obtain curcumin-loaded siRNPs (CUR@siRNPs).

The enhanced structure of CUR@siRNPs had a solubility of 1 mg/mL, significantly improving the solubility of curcumin in the body, which initially only had a solubility of <8 μg/mL, leaving it primarily insoluble. Additionally, the anti-inflammatory activity was investigated based on the presence of NO as a pro-inflammatory biomarker as a result of activated macrophages. Both free curcumin and CUR@siRNPs showed the ability to suppress the NO levels in LPS-activated macrophages; however, the enhanced CUR@siRNPs showed significantly better inhibition results compared to free curcumin.

Additionally, it was also found that the area under the curve (AUC) of free curcumin, which indicated the level of internalization and absorption into the bloodstream, stood at 1:34 ± 0:09 μg:h/mL, whereas the AUC value for CUR@siRNPs was found to be significantly greater at 2:79 ± 0:11 μg:h/mL [93]. This indicated that the concentration of drugs in the body over a prolonged period of time was improved in the CUR@siRNP model. The results also indicated that the maximum observed plasma concentration (Cmax) and the time taken to reach it (Tmax) were achieved at 0.5 h for both the free curcumin and CUR@siRNPs [93]. However, the concentration for free curcumin was only 0:69 ± 0:14 μg/mL, whereas the concentration for CUR@siRNPs was very much higher at the value of 1:74 ± 0:078 μg/mL, suggesting that the use of the NPs improved the absorption of curcumin into the bloodstream [93].

The results also highlighted that the elimination and clearance rates of the drug were improved in the CUR@siRNP model in comparison to the free curcumin [90]. This was observed as the curcumin content in the NPs was eliminated at a much slower rate compared to free curcumin [93]. The clearance rate, on the other hand, showed 18:77 ± 0:98 (mL/h) for the free-curcumin-treated mice, indicating fast removal from the body. In contrast, the CUR@siRNPs had a clearance amount of 8:98 ± 0:34 (mL/h), which was considerably much less over a longer period of time [93]. This showed that the curcumin stayed in the body for a longer period of time, thus allowing the drug to have a prolonged effect. Overall, the CUR@siRNPs improved the available amount of curcumin in the blood after oral administration, as well as allowed a prolonged retention time for enhanced future therapeutic treatments [93].

### 3.3. Cinnamaldehyde

Cinnamon is a well-known spice distributed mainly in Asia, South America, and the Caribbean. One of the most common methods of using cinnamon is in the form of cinnamon oil, which is usually diluted [94]. The oil is usually made up of the bark of the plant rather than the leaves of the tree [95]. Cinnamon is a commonly used spice for natural flavorings and fragrance additives in food, beverages, and perfumes. It has also always played an important role in the traditional medicine field, being used as a folk therapy to relieve fever, arthritis, cold, gout, and general nerve pain [96] while currently finding its way into modern medicine. Some of the modern medicine usages of this compound are in inflammatory diseases, arrhythmia, ischemia, and so on [97,98]. There have been studies on the investigation of cinnamon’s anti-inflammatory activity in aging rats, dopaminergic degeneration in mice, endothelial cells, BV2 microglia, monocytes, and macrophages [99,100,101,102], as well as in animal models of brain ischemia [103] and neuroinflammation [100].

Some of the main components of cinnamon are cinnamaldehyde (comprising around 65–80%), trans-cinnamic acid (making up about 5–10%), and eugenol (making up 4–10%). Additionally, other components of cinnamon found in smaller amounts include phenolic acids, proanthocyanidins [104], cinnamyl alcohol, terpenes, carbohydrates, coumarin, and tannins [94]. One of the major functional components of cinnamon is cinnamaldehyde, which is also found in blueberries and cranberries. In the pharmaceutical industry, the extract that holds high potential as a drug is the cinnamaldehyde, which has several beneficial characteristics, including treatment of the common cold, prevention of nausea, chronic gastrointestinal disorder, cardiovascular diseases, and others [105]. Trans-cinnamaldehyde (TCA) has been found to be the major active compound in this plant, as it possesses antineuroinflammatory, anticancer, antiallergic, antidiabetic, antifungal, anti-obesity, and other benefits [106,107,108,109]. TCA is a potent anti-inflammatory compound with effects on the activation of cells involved in inflammatory disease or injury.

TCA has the capability of inhibiting the inflammatory product of TNF-α [110], as well as IL-1β, by suppressing the NF-κB and p38-JNK pathways and MAPK [111]. Additionally, TCA’s anti-inflammatory properties are aided by its ability to suppress the expression of NO, as well as the suppression of TLR4 receptor dimerization [112]. The relationship between the two is such that TLR2 and TLR4 can sense pathogen-associated molecular patterns (PAMPs) and damage-associated molecular patterns (DAMPs). After the activation of toll-like receptors, there is activation of pro-inflammatory factors, such as IL-1β and TNF-α; the generation of ROS and reactive nitrogen species (RNS); and the release of DAMPs [105].

In a study, the anti-inflammatory effects of TCA on LPS-stimulated macrophages and the potential mechanisms by which TCA regulates NO production were investigated [113]. TCA was able to regulate the expression of pro-inflammatory cytokines, including the IL-1β, TNF-α, and IL-6 in LPS-activated macrophages [113]. The results in this study suggest that TCA significantly reduced LPS-induced NO production in a dose-dependent manner [113]. Additionally, TCA treatment reduced the mRNA expression, as well as the protein expression of iNOS, in LPS-stimulated macrophages in a dose-dependent manner. Moreover, TCA was also able to reduce the secretion of IL-1β, TNF-α, and IL-6 in LPS-activated macrophages. Overall, TCA was successfully able to exhibit anti-inflammatory properties by inhibiting and suppressing several aspects, such as the JNK, ERK, and p38 MPAKs phosphorylation in the cells [113].

When it comes to bioavailability from intravenous administration, cinnamaldehyde is absorbed rapidly in the gut, followed by metabolization and excretion through the urine. In one study, the blood concentration of this extract showed a decreasing profile in a biphasic manner. This is mainly because a large amount of cinnamaldehyde is oxidized to cinnamic acid within 30 min, which occurs mainly in the liver, stomach, and small intestine. The half-life of cinnamaldehyde is 1.7 h, which means there is quick removal of the cinnamaldehyde from the body over a short period of time [114]. In another study, the oral bioavailability of cinnamaldehyde was approximatively 20%, with a distribution of its metabolites throughout the body [115].

#### Cinnamaldehyde Delivery Using Nanoparticles

A study using a whey protein isolate–dextran conjugate with chondroitin sulfate was conducted to prepare a biopolymer nanoparticle that could protect cinnamaldehyde in harsh environments. These harsh environments included extreme pH, high ionic strength, and high temperature. Cinnamaldehyde-loaded nanoparticles (NPs-C) were prepared with a high encapsulation efficiency and a loading capacity of 76.57% and 19.02, respectively. They were sized at 185 nm and had a PDI of 0.22. The results showed that the NPs-C were stable over a pH range of 1–10 and at varying ionic strengths, as well as heating at 90 °C for 30 min. There was improved stability when the NPs-C were stored for 6 weeks at 4 °C, as well as at 25 °C. Lastly, the results also indicated a sustained microbial activity, which signifies drug release over a prolonged period of time compared to free cinnamaldehyde. Overall, this combination of NPs-C showed promising prospects, as it was able to withstand the harsh conditions of the body while also having the favored characteristic of sustained release [116].

Another study tested the characteristics of a cross-linked chitosan and cinnamaldehyde nanoparticle. The chitosan nanoparticle was produced from an ionic cross-link of biopolymer chitosan with sodium tripolyphosphate (TPP). The product was structurally stable and had amorphous characteristics. It also showed enhanced antimicrobial activity in comparison to free chitosan and cinnamaldehyde and was effective for both Gram-positive and Gram-negative bacteria. The enhanced antimicrobial properties were contributed by the larger surface area available to interact with the bacterial cells due to its nano-size ranging from 80–150 nm. Although this product was not focused on pharmaceutical use, it provided enough information to confirm the enhanced effects of nanoparticles on natural functional food [117].

### 3.4. Eugenol

Eugenol is a phenolic functional food sourced from the phenylpropanoid compound [118] found in various foods, such as coffee, soybeans [119], honey, bananas [120] cloves, oregano [121], cinnamon, basil [122], and bay leaves [123], and it presents itself as a yellow viscous oil at room temperature. Functional foods containing eugenol are commonly used as flavoring agents for various types of food recipes, as well as cosmetics [124]. Its scientific name is 2-methoxy-4-prop-2-enylphenol, and it is an allylbenzene compound known to have several beneficial properties [14]. Some of these properties include antiseptic, antifungal [125,126], antipyretic [127], antioxidant [128], antibacterial [129], anti-inflammatory [130], antitumor [131], and analgesic properties [132], amongst others [133]. As inflammation is a complex protective response of the body against harmful agents such as microorganisms or damaged cells, eugenol’s anti-inflammatory properties have the ability to inhibit pro-inflammatory factors, including IL-1β, IL-6, TNF-α, PGE2, iNOS, COX-2, NF-κB, and 5-lipoxygenase (5-LOX) [134]. One of the main inflammatory pathways is the excessive production of prostaglandins. COX is the key enzyme required in the metabolization of arachidonic acid to prostaglandins [135]. Additionally, lipoxygenases (LOXs) are enzymes that speed up the peroxidation of polyunsaturated fatty acids [135]. The 5-LOX enzyme is specifically associated with inflammation, bronchoconstriction, asthma, anaphylaxis, and hypersensitivity [136,137].

In another study, eugenol showed the ability to block the cyclooxygenase hydrophobic channel by means of hydrophobic H-bond interactions with the fragment Ser 530 and hydrophobic interactions with Tyr 385. Eugenol demonstrated the ability to interact strongly with the amino acid Ser 530, as well as Tyr 385, through hydrophobic interactions. Ser 530 and Tyr 385 are important for the inhibition of COX-2 by several compounds. Ser 530 has also been shown to influence stereochemistry for the addition of oxygen to the prostaglandin product. Additionally, the catalytic residue Tyr 385 is responsible for the conversion of arachidonic acid to prostaglandin G2 by the transfer of an electron to the heme from Tyr 385 of the protein generated to the tyrosyl radical in the cyclooxygenase active site. Eugenol was also shown to inhibit arachidonic acid catalysis in prostaglandin G2 directly in the active pocket site at the end of the hydrophobic channel of COX by H-bond interaction with Ser 530 [138].

#### Eugenol Delivery Using Nanoparticles

Despite the medicinal benefits, eugenol has some limitations. For example, in a study on the application of eugenol on the skin, eugenol was found to be volatile and had an irritant effect on the skin that may have been caused by the absence of efficacy, thus also limiting its topical use. To overcome this limitation, polymeric nanocarriers were used to deliver the eugenol. The treatment of eugenol alone at various concentrations did not reduce the thickness of an ear edema (0.29 ± 0.01 at 0.04 mg/ear, 0.29 ± 0.02 at 0.08 mg/ear, and 0.33 ± 0.01 at 0.16 mg/ear) when compared to the control group (0.32 ± 0.03). When the eugenol was delivered in a nanocapsule suspension, the ear edema was significantly reduced (0.10 ± 0.009 at 0.08 mg/ear and 0.08 ± 0.008 at 0.16 mg/ear) when compared to the control group of blank nanocapsule suspensions (0.22 ± 0.022). The results indicate that nanocarriers provided a controlled drug release when used topically, as well as improved efficacy when used as treatment. Overall, the irritant effect of pure eugenol observed may have been related to the anti-inflammatory activity and drug control on skin permeation [139].

In another similar study, a nanoemulsion was used to evaluate the anti-inflammatory effects of eugenol via topical delivery. Nanoemulsions are transparent or translucent dispersions of oil and water that are stabilized by surfactant and cosurfactant molecules of less than 100 nm in size that allow enhanced transdermal permeation. Eugenol was found to be safe for topical application up to a concentration of 5%; however, it tended to show local irritation when reaching a concentration of 8%. The results indicate that the nanoemulsion formulations of eugenol at 1%, 2%, and 4% concentrations showed superior anti-inflammatory effects compared to eugenol on its own. The ideal formulation was identified at 2% eugenol (oil phase), 14% Tween 20 (surfactant), and 14% isopropyl alcohol (cosurfactant) in water. This provided a nanoemulsion with a PDI of 0.3 and a median droplet diameter of 24.4 nm (d50). The animal studies also showed that, after 1.5 h of the treatment with nanoemulsion, increased anti-inflammatory activity was observed compared to piroxicam gel, which is a commercially available product. The researchers conducted further analysis and found that the nanoemulsion containing piroxicam had lower anti-inflammatory activity in comparison to piroxicam on its own, and this may have been caused by the decreased stability and the increased particle size. Overall, nanoformulations of eugenol have potential for their use against various inflammatory diseases [140].

### 3.5. Naringenin

Naringenin is a naturally occurring flavanone and can be found in citrus fruits and vegetables, such as lemon, grapefruit, tomatoes, and oranges [141]. This functional food has the chemical name of 2,3-dihydro-5,7-dihydroxy-2-(4-hydroxyphenyl)- 4H-1-benzopyran-4-one and a molecular weight of 272.26 (C15H12O5) [142]. Naringenin is a flavanone that is derived from naringin or narirutin hydrolysis. In addition, naringenin strategically occupies a central position as the primary C15 intermediate in the flavonoid biosynthesis pathway [143]. The metabolic pathway for this compound constitutes a six-step process, successively catalyzed by phenylalanine ammonia lyase (PAL), cinnamate 4-hydroxylase and its associated cytochrome P450 reductase, para-coumarate-CoA ligase, chalcone synthase (which plays the role of the key enzyme for the synthesis of naringenin), and chalcone isomerase [144]. Naringenin is insoluble in water but soluble in organic solvents, which can prove to be a limitation to its usage.

There is growing evidence from in vitro and in vivo animal studies that this functional food has beneficial effects, including hepatoprotective, anti-atherogenic, anti-inflammatory, antimutagenic, anticancer, and antimicrobial properties, even suggesting its applications in cardiovascular, gastrointestinal, neurological, and metabolic disease control and management [145,146,147]. There is potential for its use in a wide variety of diseases, such as chronic airway diseases, liver diseases, lung diseases, cancer, cardiovascular diseases, and many more [142].

Naringenin has shown potential therapeutic benefits in several models of inflammatory pain. Naringenin has the potential to inhibit pain-like behavior caused by inflammatory stimuli, which include phenyl-p-benzoquinone, acetic acid, formalin, complete Freund’s adjuvant, capsaicin, carrageenan [148], superoxide anions [149], and LPS [150]. Usually, PAMPs and DAMPs are related with molecular patterns and inflammatory mediators, which in turn activate resident macrophages that produce chemotactic molecules. These chemotactic molecules recruit leukocytes, such as neutrophils, to the inflammatory area during the initial stage of inflammation. Once the macrophages and neutrophils are activated, they induce oxidative stress by producing superoxide anions and other ROS and RNS.

Naringenin can inhibit leukocyte recruitment and the production of superoxide anions and can increase antioxidant capacity by upregulating the antioxidant glutathione (GSH) [148,149,151]. On the other hand, naringenin also can induce Nrf2 activation in macrophages and trigger antioxidant and anti-inflammatory responses [149]. PAMPs, DAMPs, and ROS induce NFκB activation in macrophages, resulting in the production of prohyperalgesic cytokines, such as TNFα, IL-1β, and IL-6. These prohyperalgesic cytokines then induce the production of lipid mediators, such as PGE2, that sensitize the nociceptor neurons. Naringenin was shown to inhibit LPS-induced NFκB activation in vivo and in vitro, which contributed to the inhibition of IL-33, TNF-α, IL-1β, and IL-6 production and the downregulation of COX2 mRNA expression, as described in the pathway mechanism below [152].

Although it has therapeutic potential, naringenin has its limitations, such as being a crystalline and hydrophobic compound with low oral bioavailability [153]. Thus, naringenin tends to have slower dissolution when used in the form of oral dosages. Additionally, it has poor ability to cross biological membranes. All these factors limit the therapeutic usage of naringenin [154].

#### Naringenin Delivery Using Nanoparticles

To enhance the solubility and cellular uptake, naringenin was loaded into liquid crystalline nanoparticles (LCNs) in a study. The naringenin-loaded LCNs had a size of 175.9 ± 2.7 nm, a PDI of less than 0.2, a zeta potential of −23.8 ± 1.03 mV, and an entrapment efficiency of 78.56 ± 0.13%. PDI is an indicator of the homogeneity of the size distribution, and the narrow size distribution and uniform size confirmed the homogeneous nature of the formulation. This formulation showed an initial burst of release in the first two hours, followed by a 9 h long sustained release. This yielded a total 57.85% release of naringenin from the formulation [152]. In the same study, the anti-inflammatory effects of naringenin-loaded LCNs were studied. The results indicated that the pro-inflammatory factors, including IL-1B, TNF-a, IL-6, and IL-8, were sufficiently reduced by naringenin-loaded LCNs in comparison to the blank LCNs [155].

In another study, naringenin was loaded into solid liquid nanoparticles (SLNs). SLNs have added benefits of low toxicity, controlled drug release, reduced chemical degradation, targeting options, large-scale production, increased physical stability, and low cost [156]. In this study, naringenin-loaded SLNs had a size of 98 ± 0.61 nm, a PDI of 0.258 ± 0.058, and a zeta potential of −31.4 ± 0.98 mV. The zeta potential indicated that this colloidal dispersion was a system that was physically stable. Additionally, it was also found that the naringenin plasma concentration was substantially higher in rats that were treated with naringenin-loaded SLNs in comparison to naringenin alone [157], which may have been due to the smaller particle size [158]. Additionally, it was also seen that there was a significantly prolonged half-life and mean residence time of the naringenin-loaded SLNs in comparison to free naringenin [157]. This may have been due to the drug being embedded into the SLN matrix, which prevented it from enzymatic degradation. Overall, naringenin successfully exhibited sustained release and prolonged circulation in the blood when released from its SLNs [159], allowing it to be considered as a potential clinical treatment.

### 3.6. Capsaicin

Capsaicin, chemically 8-methyl-N-vanillyl-6-nonenamide, is a common lipid-soluble compound found in various *Capsicum* species, known as chilis and hot peppers [160]. Capsicum has been used in the human diet since 7500 BC. Capsicum belongs to the Solanaceae family, which has over 200 varieties, and it varies in size, shape, flavor, and sensory heat. The most common species studied are the *Capsicum annuum*, *Capsicum baccatum*, *Capsicum chinense*, *Capsicum frutescens*, and *Capsicum pubescens* [161], which are known for their burning and irritant effect. The rather pungent capsicum has been used for generations as a spice or as food. However, the effects of capsaicin go well beyond the taste, and its role in plants’ health helps us to understand how its use can improve human health.

These peppers are usually found in the tropical and humid areas of Central and South America [162] and are used for folk remedies, including dropsy, colic diarrhea, asthma, arthritis, muscle cramps, and toothaches [161]. Additional uses of capsaicin in medical traditions are rooted various cultures, including Indian, Native American, and Chinese, for many conditions, including arthritis, rheumatism, skin rashes, stomach pain, dog or snake bites, and wounds [163]. The modern therapeutic uses for capsaicin are for diseases such as cancer and obesity, as well as cardiovascular and dermatological diseases [164,165]. The ability of the lipophilic alkaloid capsaicin to act as a therapeutic agent is due to its various properties, including its analgesic [166], antioxidant [167], anticancer [168], anti-obesity [169], and anti-inflammatory [170] properties, which have been used over the years in clinical practice. One of the main reasons as to why capsaicin is widely studied and used is due to its chemical structure that allows it to be well-absorbed by the body when administered topically and orally, with absorption levels up to 94% [171].

In one study, the antioxidant and anti-inflammatory activities of pepper leaves and pepper fruits were evaluated. The pepper leaves were more effective than pepper fruits in increasing radical scavenging activity and ameliorating inflammatory responses in LPS-induced RAW 264.7 cells by inhibiting the production of several factors, including IL-6, NO, and TNF-α. Additionally, the production of ROS by LPS-activated macrophages was significantly reduced by the pepper leaves, which directly indicated the antioxidant ability of this functional food [172].

#### Capsaicin Delivery Using Nanoparticles

Capsaicin exerts its anti-inflammatory activities by inhibiting the production of pro-inflammatory factors, including COX-2, PGE2, and iNOS [173]. When capsaicin was used in topical formulations it showed limitations, including having restrictions on the skin due to its lipophilic drug characteristics, as well as having side effects such as skin irritation. Hence, a more innovative delivery system of capsaicin using nanoemulsions has been developed, as it possesses many benefits, including small size, high drug-loading capacity, and easy manufacturing [174]. These nanoemulsions are made up of oil, a surfactant, cosurfactants, and water.

A nanoemulsion delivery system can prove to be beneficial as it can disperse hydrophobic compounds in aqueous media and enhance the permeability of capsaicin across the skin without any side effects of skin irritation [175]. Additionally, nanoemulsions can be formulated into gels or creams due to their low viscosity, which in turn can improve the dermal and transdermal delivery of capsaicin [176,177,178]. This is due to the large surface area and low surface tension of the small oil droplets, which result in better interaction with the skin and allow improved penetration of the active compounds. The surfactants in nanoemulsion formulations can interfere with the lipid structure of the skin, thus enhancing the topical penetration of the drug [179]. The results from a study indicated that a nanoemulsion formulation in the form of topical gel was able to enhance the analgesic and anti-inflammatory effects of capsaicin compared to a previous commercial formulation owing to its enhanced skin permeability, as well as being nano-sized particles [180]

In another study, the optimum conditions of capsaicin-loaded nanoemulsion formulations were evaluated for topical application. The transdermal delivery route was preferred over the oral drug delivery route for capsaicin due to its avoidance of hepatic first-pass metabolism, reduction of side effects, and improvement in drug solubility [181]. However, the challenge of transdermal drug delivery is to surpass the stratum corneum, which has hydrophobic characteristics and, therefore, is not favorable for hydrophobic drugs [182]. In order to overcome this limitation, various nanotechnology-based delivery systems have been studied for improved transdermal delivery.

Biocompatibility, biodegradability, the ability to sustain the active ingredient until it reaches the target site, and easy excretion from the body after a certain duration are some of the important requirements for achieving an effective drug delivery system [181]. The most suitable delivery system with these requirements is the nanoemulsion, as it has a high thermodynamic stability, small droplet size, and—most importantly—enhanced solubilization for hydrophobic ingredients, which together allow easy penetration across the skin surface. A capsaicin nanoemulsion was able to successfully permeate across all layers of the skin, including the stratum corneum up to the dermis [183].

### 3.7. Boswellic Acids

Boswellic acids (BAs) are a group of compounds sourced from the *Boswellia* species of the Burseraceae family, with its more common variants known as *Boswellia serrata* (BS) and *Boswellia dalzielii* (BD). They have been used for over centuries in traditional medicine as many forms of treatment, as well as for ceremonial purposes. BAs are sourced from a moderate- to large-sized tree that grows in the mountain areas of geographic locations including India, northern Africa, and the Middle East [184]. This plant produces a milky fluid with a whitish color called oleogum resin or frankincense, which is exudated from the tree bark and functions to protect the trees from pest and infections. The oleogum resin, which is present as a milky fluid, eventually solidifies when exposed to air, and often the resin component of this plant consists of BAs, terpenes, and terpenoids.

BAs are pentacyclic triterpenic acids that carry many beneficial properties and can potentially be used to treat many diseases. There are various forms of different pentacyclic triterpenic acids found in the resin, some of which were identified and listed by Büchele and Simmet [185] and are listed in Table 1. However, the most active BAs are the 11-Keto-β-boswellic acid (KBA) and the 3-Acetyl-11-keto-β-boswellic acid (AKBA), the molecular structures of which are shown in Table 2. Over several years of scientific studies, it has been found that BAs consist of several useful properties, including anti-inflammatory, antifibrotic [186], tumor suppressor [187], antimicrobial, antioxidant [188], and immunomodulatory activities [189], as well as having ability to improve learning and memory in neurological diseases [190]. The anticancer properties of BAs are contributed by targeting important cell-signaling components, such as MAPK, NF-κB, TNF-α, and ERK1/2, thus regulating cell proliferation, metastasis, invasion, and migration [191,192].

The anti-inflammatory effects of BAs are mainly contributed by suppressing leukotriene synthesis via the inhibition of 5-LOX [193], which in turn increases cell permeability. BAs also inhibit leukocyte elastase, which is required for their anti-inflammatory activities. Additionally, BAs can specifically inhibit the activity of 5-LOX in a non-redox-dependent manner without affecting the activities of COX or 12-LOX [194]. In addition, AKBA was shown to be a natural inhibitor of the NF-κB transcription factor, which is well-known to be a crucial downstream mediator for the production of pro-inflammatory cytokines in the process of inflammation [195].

#### Boswellic Acid Delivery Using Nanoparticles

Although BAs have many beneficial properties, their pharmacological activity is limited by several issues, such as having low oral bioavailability and poor aqueous solubility. The use of nanoparticles may be able to overcome most of their limitations. In one study, the anti-inflammatory activity of KBA was studied using a poly-DL-lactide-co-glycolide-based nanoparticle formulation. When the KBA was administered as a KBA-NPs formulation, a rat paw edema was inhibited by 61.5% at 5 h of administration in comparison to 34.9% when treated with KBA alone. Overall, the results indicate that there was a 7× improvement in oral bioavailability for KBA-NPs compared to KBA alone. Additionally, there was also an increase in the in vivo anti-inflammatory activity by 1.7× when using the KBA-NP treatment instead of just KBA alone [196].

In another study, silver nanoparticles (AgNPs) were used to deliver BAs, and their anti-inflammatory activity was tested on rat paw edema. The BA-AgNPs showed significant anti-inflammatory activity in comparison to raw BAs at 500 mg/kg and 2000 mg/kg dosages at different intervals of 30, 60, 120, and 180 min with no signs of mortality or toxicity. BA-AgNPs showed better characteristics in terms of sustained release in comparison to BAs alone. These results showed the release of BA alone being 99 ± 0.2% in 10 h, while the BA-AgNPs had a release of 98.4 ± 0.4% at 24 h. The results for the BA-AgNPs, which had an R2 value of 0.89, suggest that this formulation fell under the super case II transport mechanism category according to Korsmeyer–Peppas, as it had both first-order and sustained release behavior [197].

Another study evaluating the antiproliferative effects of BAs on chitosan NPs (CNPs) on a lung cancer cell line showed better and beneficial outcomes of BA-CNPs in comparison to the BAs. The entrapment efficiency of the BA-CNPs was 80 ± 0.48% with sustained intercellular reservation, which was superior compared to the free drug on its own. BA-CNPs showed superior cytotoxic and anti-proliferative activities via apoptosis when compared to BAs alone. The IC50 value was greater for BA-CNPs (29.59 µM) compared to the free drug (17.29 µM) [198].

### 3.8. Rutin

Bioflavonoids have been increasingly studied and used in healthcare, as they have the potential for a wide range of biological and pharmacological benefits, being much safer to use, as well as having lower cost of production. Rutin is a flavonol glycoside found in several species of plants and fruits, such as apples, flowers of the pagoda tree, passionflowers, tea berries, and buckwheat. This natural component is also known by several other names, such as rutoside, quercetin-3-rutinoside, sophorin, which is more commonly found in buckwheat. The name rutin originates from the plant *Ruta graveolens*, which contains the rutin compound. The chemical name of rutin is 3,3′,4′,5,7-pentahydroxyflavone-3-rhamnoglucoside, and is has a structure consisting of a flavonol aglycone quercetin that is bound with a disaccharide rhamnosyl glucose [199]. Rutin has proved to possess anti-inflammatory [200], antitumor [201], neuroprotective [202], antimicrobial [203], antiviral [204] analgesic [205], and anti-oxidant [206] properties.

One of the most useful properties of rutin is its ability to suppress the production of pro-inflammatory factors. In a study, rutin showed a reduced production of TNF-α and IL-1β production, which are very common pro-inflammatory factors in the microglia in Alzheimer’s disease. Rutin also decreased the production of ROS, signifying potential therapeutic benefit against the disease [207]. In another study, the neuroprotective activity of rutin was evaluated on intracerebroventricular-streptozotocin (ICV-STZ)-infused rats. Rutin was able to diminish the STZ-induced inflammation by reducing COX-2, IL-8, NF-κB, and NO, which are all major contributors to inflammation. Overall, rutin was able to prevent major changes in the hippocampi of rats and, therefore, is potentially beneficial in preventing cognitive loss [202].

In another study on ALI, a pretreatment of rutin inhibited the histopathological changes, as well as the infiltration of polymorphonuclear granulocytes into bronchoalveolar lavage fluid, in LPS-induced ALI. Rutin also suppressed the production of pro-inflammatory cytokines and lipid peroxidation in a concentration-dependent manner. It is important to note that rutin was able subdue the phosphorylation of NF-κB and MAPK and the degradation of Iκβ, an NF-κB inhibitor, which are important components of the inflammation process [208]. Rutin administration (300 mg/kg) also showed a significant decrease in the numbers of lymphocytes, neutrophils, leukocytes, and macrophages in the bronchoalveolar lavage fluid of a mouse model of cigarette-smoke-induced COPD. Additionally, rutin was also capable of reducing the expression of pro-inflammatory factors TNF-α, IL-8, and PAF in a mouse model exposed to cigarette smoke [209].

#### Rutin Delivery Using Nanoparticles

Although rutin has shown many beneficial properties, it still has its limitation when considering its expansion into pharmaceutical applications. The main limitations of rutin are low water solubility and low oral bioavailability [210]. An upcoming way to overcome this limitation to reaching the target site of the lungs is to use the delivery method of nanoparticles. In one study using nanoparticles, LCNs were used to increase the drug bioavailability and stability while also reducing the overall toxicity of rutin in the inhibition of in vitro cancer proliferation. Rutin-LCNs showed an overall encapsulation efficiency of 70% with a sustained release of rutin for over 24 h. Rutin-LCNs significantly reduced the cell proliferation in a concentration-dependent manner. Thus, rutin showed better oral bioavailability and water solubility in its nanocarrier formulation, suggesting its potential use as a therapeutic agent [211].

In another study, rutin-conjugated gold nanoparticles decreased collagen-induced arthritis in rats. Rheumatoid arthritis (RA) is an autoimmune disorder related to inflammation where increased amounts of free NO, PO, and superoxide anion radicals are observed. Additionally, the inflammatory response of the NF-kB regulator is expected to lead to the production of COX-2, MMPs, iNOS, and pro-inflammatory cytokines, as well as chemokines [10]. In this study, rutin-AuNPs substantially decreased NO and PO amounts compared to pure rutin and its control. There was also a greater decrease in the expression of NF-kB and iNOS in the rutin-AuNP-treated group, suggesting its overall potential as a therapy against inflammation [212].

### 3.9. Neem Compounds

The neem plant, *Azadirachta indica*, has been widely used through the ages in medical folklore. Various parts of the neem plant, including the leaves, bark, fruit, flower, oil, and gum, have been used for various medical purposes, including cancer, hypertension, heart diseases, and even diabetes. This plant is a tetranortriterpenoid [213] and a member of the Meliaceae family, which is mainly cultivated in the southern parts of Africa and Asia, including India, Pakistan, Bangladesh, and Nepal [214]. One of the most important active constituents of neem is azadirachtin. However, there are other crucial active compounds, including nimbolinin, nimbin, nimbidin, nimbidol, sodium nimbinate, gedunin, salannin, and quercetin. The leaves of the neem plant contain nimbin, nimbanene, 6-desacetylnimbinene, nimbandiol, nimbolide, ascorbic acid, n-hexacosanol and amino acid, 7-desacetyl-7- benzoylazadiradione, 7-desacetyl-7-benzoylgedunin, 17-hydroxyazadiradione, and nimbiol [215].

Neem has various pharmaceutical properties, including antibacterial, antifungal, free radical-scavenging, detoxification, anti-inflammatory, hepatoprotective, chemopreventive, DNA repairing, cell-cycle alteration, programmed cell death mitigation autophagy, anti-angiogenic, and chemotherapeutic properties, amongst others [216,217,218,219,220]. However, the exact molecular mechanism in the prevention of pathogeneses of diseases is not understood. Neem exerts its antimicrobial activity through an inhibitory effect on microbial growth and the potentiality of cell-wall breakdown [221]. Additionally, neem has free-radical-scavenging properties, as it is a rich source of antioxidants. Other components of neem, such as azadirachtin and nimbolide, have shown concentration-dependent antiradical-scavenging activity and reductive potential [222]. Neem has the ability to manage cancer through the regulation of various tumor suppressor genes (e.g., p53 and pTEN), angiogenesis (VEGF), transcription factors (e.g., NF-κB), and apoptosis (e.g., bcl2 and bax). Neem also functions as an anti-inflammatory agent via the regulation of pro-inflammatory enzyme activities, including COX and LOX enzymes [214].

One of the most useful properties of neem extract is its anti-inflammatory property [223]. Inflammation can be identified as a pathophysiological condition in a wide range of diseases, including COPD, cancer, diabetes, and many others. The main bioactive compound in neem associated to its anti-inflammatory property is limonoid. Limonoid is a furanolactone that has the ability to inhibit the production of inflammatory mediators, as well as being able to act as a pain anesthetizer [224]. Limonoid has an inhibitory effect over inflammatory molecules, including TNF-α and interleukins [225,226]. Another active compound that contributes to the anti-inflammatory effect of the neem plant is epoxyazadiradione. Neem extract shows cytotoxic abilities through the modulation of macrophage inhibitory factors, as well as the inhibition of NF-kβ. The inhibition of NF-kβ blocks the production of pro-inflammatory cytokines, including IL-1α, IL-1β, IL-6, and TNF-α [227].

The progression of inflammation results in activation of the COX pathway. Neem extract may inhibit COX1 and COX2 [228]. The triterpenes found in neem extract have the ability to modulate the metabolism of prostaglandin and thromboxane production, which are important for the conversion of arachidonic acid to PGH2 and later to PGE2, all of which are mediated by COX2 [229]. Neem extract has been shown to disrupt IL-1 and COX2 stimulation, resulting in an antipyretic effect while also inhibiting the nuclear translocation of NF-kβ. The inhibition of NF-kβ, in turn, reduces the activation of cytokines and TNF-α, resulting in lowering of inflammation in the body [227].

In another study conducted on dextran-sodium-sulfate (DSS)-induced colitis in rats, the effects of neem leaf extracts on the levels of IL-6 and TNF-α were evaluated. The results indicated that TNF-α and IL-6 expressions decreased in all groups receiving neem treatment. This confirmed that neem did, in fact, show anti-inflammatory properties [230], as IL-6 and TNF-α are directly associated to the instant triggering of inflammation, and this same concept can be applied for the use of neem in COPD patients.

Although these studies were not focused on COPD, the inflammatory pathway is similar to that observed in general inflammation, including COPD. Additionally, crude neem as a natural product does have its limitations, including photosensitivity and rapid degradation resulting in lower efficiency [231]. One way of overcoming these limitations is by using a better carrier system in the form of nanoparticles. Using nanoparticles as a carrier can aid in modifying the release profile of the extract, which can in turn enhance the anti-inflammatory property, as well as stability, of the formulation [232].

#### Neem Compound Delivery Using Nanoparticles

In one study, the antibacterial activity of neem extract was tested as a nanoemulsion formulation. The droplet size that was found to be the most effective ranged from 30–70 nm and had a spherical shape. Additionally, the researchers used Tween 20 as a suitable surfactant, as it has low sensitivity to pH, is nontoxic, and is biocompatible [216].

In another study, a formulation of *A. indica* in silver nanoparticles was used to characterize and test its larvicidal activity. It is a well-studied application that silver nanoparticles show their beneficial characteristics for their antibacterial, antiviral, and antifungal activities [233]. In this study, the size distribution of the silver nanoparticles ranged from 12–24 nm, with an average size of 17 ± 4 nm. The results also indicated the presence of other components, including oxygen and carbon. These metabolites have a crucial role of acting as stabilizing agents during the synthesis of silver nanoparticles. This was performed when they surrounded and began to develop a thin, capping layer of organic molecules that, in the long run, posed to be more cost-effective, as well as reduced the toxicity due the added stability [234]. Additionally, the results showed a concentration-dependent larvicidal activity, suggesting the possible and potential use of this formulation for antilarval applications in medicine and pharmaceuticals [235].

## 4. Clinical Trials on Nanotechnology-Based Drug Delivery Containing Functional Foods

Several notable clinical studies and trials have been conducted recently on potential nanotechnology-based drug delivery containing beneficial functional foods, especially targeting neurological and also certain lung diseases like COPD. These have been highlighted in Table 3 and Table 4.

## 5. Future Prospects

Functional foods possess many beneficial properties and medical purposes that can play a crucial role in the wellbeing of mankind. Nutrient-based therapies are on the rise, and functional foods have been widely studied also in respiratory disease conditions. There are already a handful of clinical studies that have been conducted to assess the potentials of such functional foods in lung diseases. However, to meet the future demands for these functional foods, the food and pharmaceutical industries must take note and address several crucial challenges of low bioavailability, establishing optimal intake levels, lack of solubility, developing adequate food-delivery matrices, product formulation, and regulatory challenges. Other hazardous aspects that need to be addressed are the potential bioaccumulation, reduced excretion, or even toxic side effects of functional foods that can be detrimental to human health.

In the near future, functional delivery systems using nanoparticles may be further studied in order to protect potent food compounds and extracts from harsh food processes and storage conditions. There is also a need for improvements in the technology used to manufacture these products, as that is crucial in increasing the efficacy of the bioactive compounds found in functional foods. Additionally, delivery systems using nanoparticles can potentially improve the bioavailability of the extracts, as well as enhance the absorption of bioactive molecules into the body. Nanotechnology can also allow functional foods to be used in a target-specific method to ensure that a higher percentage of the active compound reaches the appropriate regions and target cells in the body. Lastly, there needs to be a better understanding of each functional food, such as the rate of digestion and metabolism, as well as the ability to penetrate biological barriers. The use of safe nanocarriers with functional food may allow slower metabolism while simultaneously providing sustained release of the extract. This would then allow the therapeutic usage of functional foods to be effective for a prolonged period of time.

## 6. Conclusions

In conclusion, the industry of functional foods has yet to see much advancement with the addition of nanoparticle delivery systems. More research and clinical studies must be conducted over the next few years to ensure the enhanced effectiveness and, most importantly, the safety of these new products for human health. Some of the major considerations that research bodies and the pharmaceutical industry, in general, must look into are the possible negative effects or toxic side effects of functional foods, nanoparticle delivery systems, and the combination of both of these on the human body. Additionally, it is also important for the pharmaceutical industry to ensure safe and optimum levels of absorption so that the cost of products can be controlled, and a larger population of people may have access to it.

## Figures and Tables

**Table 1:** The various types of pentacyclic triterpenic acids in *Boswellia* species extract [185].

α-Boswellic Acid	β-Boswellic Acid
Acetyl-α-boswellic acid	Acetyl-β-boswellic acid
Lupeolic acid	11-Keto-β-boswellic acid
Acetyl-lupeolic acid	Acetyl-11-keto-β-boswellic acid
11-Dehydro-α-boswellic acid	9.11-Dehydro-β-boswellic acid
Acetyl-9-11-dehydro-α-boswellic acid	Acetyl-9-11-dehydro-β-boswellic acid

**Table 2:** Chemical structures of functional food compounds.

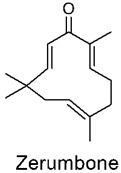	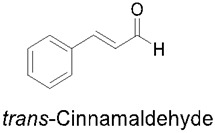
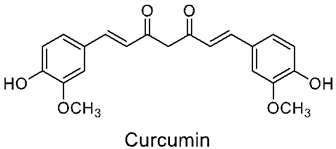 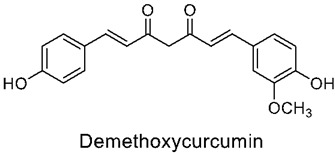 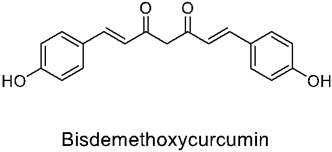	
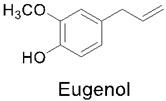	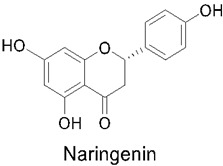
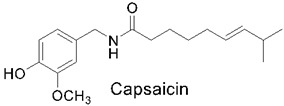	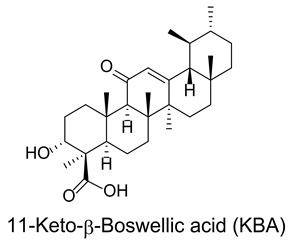 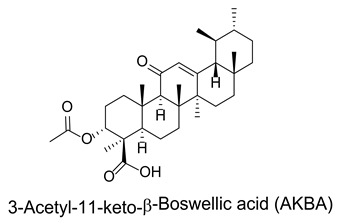
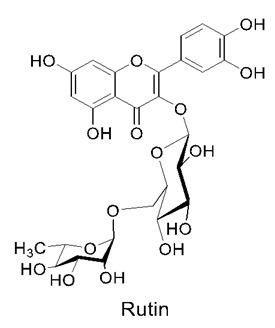	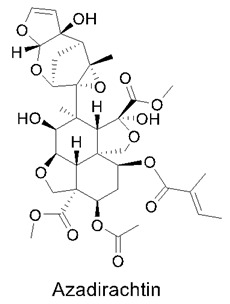 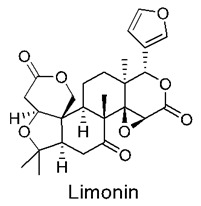 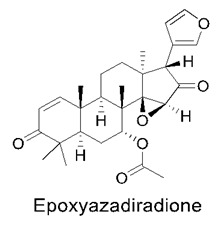

**Table 3:** Notable clinical trials involving common functional foods on various diseases.

Functional Food	Drug Delivery Involved/Type	Clinical Trial Number	Description
Curcumin	Curcumin Nanoparticles	NCT02104752	The clinical study was aimed to test if the curcumin nanoparticles were able to improve the behavioral measures, as well as act as biomarkers of cognition and neuroplasticity, in patients who had been diagnosed with schizophrenia and were already receiving a stable dose of antipsychotic [236].
Capsaicin	Capsaicin Nanoparticles	NCT01125215	This study tested the efficacy and safety of a 0.75% topical capsaicin nanoparticle preparation against a placebo for patients with painful diabetic neuropathy [237].

**Table 4:** Notable clinical studies involving functional foods for COPD and other relevant lung diseases.

Functional Food	Drug Delivery Involved/Type	Clinical Trial Number	Description
Curcumin	Daily Oral Consumption	NCT04687449	A double-blind placebo-controlled trial on 120 COPD patients who were tested at random with curcumin or a placebo for 90 days. The outcomes were compared among other study arms. No dose escalation was used [238].
Cinnamaldehyde	Inhalation	NCT03700892	This clinical research looked into identifying the effects of cinnamaldehyde-containing e-cigarettes on airway epithelial cell ciliary function, as well as airway immune cells, in humans [239].
Capsaicin	Inhalation	NCT01621685	This study tested the effects of capsaicin inhalation on human safety, specifically on young and older men who had been diagnosed with COPD [240].

## Data Availability

Not applicable.
